# Supplementary material for: Dynamic transcriptomic profiles of zebrafish gills in response to zinc supplementation
Source: BMC Genomics. 2010 Oct 11;11:553. doi: 10.1186/1471-2164-11-553 (PMC3091702; doi:10.1186/1471-2164-11-553)
Supplement: Additional file 2 — Interactive Direct Interaction Network representing the molecular interactions between zinc, copper, iron, calcium and proteins encoded by transcripts changed by zinc supplementation. Mini web-site containing index.html and hyperlinked pages in subdirectory describing a Direct Interaction Network automatically generated based on curated interactions contained within the proprietary PathwayArchitect database. Ovals represent proteins and the circles symbolize metal ions. Objects are coloured by their abundance in zebrafish at the time-point they were significantly different from the control is a scale from -4 fold (dark green) to +4 fold (dark red). Where significant differences were found at more than one time-point, the colour overlay shows expression at the first instance. Dark blue squares denote 'binding', and light blue squares 'expression'; green squares stand for 'regulation', green diamonds for 'metabolism', and green circles for 'promoter binding'. Arrow heads indicate directionality of the interaction where annotated. All nodes and edges can be further interrogated by selecting the relative area of the image. [file 1471-2164-11-553-S2.zip › PathwayArchitect Zn xs DIN/82100.html]

# PROTEIN: STMN1

|  |  |
| --- | --- |
| Name | STMN1 |
| Type | PROTEIN |
| Description | stathmin 1/oncoprotein 18 |
| Note | This gene belongs to the stathmin family of genes. It encodes a ubiquitous cytosolic phosphoprotein proposed to function as an intracellular relay integrating regulatory signals of the cellular environment. The encoded protein is involved in the regulation of the microtubule filament system by destabilizing microtubules. It prevents assembly and promotes disassembly of microtubules. Alternatively spliced transcript variants encoding the same protein have been identified. |
| Alias | stathmin |
|  | AI131641 |
|  | Lap18 |
|  | SMN |
|  | STMN1 |
|  | Oncoprotein 18 |
|  | 19K |
|  | phosphoprotein 19 |
|  | prosolin |
|  | OP18 |
|  | Prosolin |
|  | oncoprotein18 |
|  | p18 |
|  | PP19 |
|  | Lag |
|  | PP18 |
|  | op18 |
|  | metablastin |
|  | Phosphoprotein p19 |
|  | Pr22 |
|  | Leukemia-associated phosphoprotein p18 |
|  | pp17 |
|  | leukemia-associated phosphoprotein p18 |
|  | MGC72884 |
|  | Leukemia-associated cytosolic phosphoprotein stathmin |
|  | oncoprotein 18 |
|  | pig |
|  | Stmn1 |
|  | PR22 |
|  | Leukemia-associated gene protein |
|  | leukemia associated phosphoprotein p18 |
|  | PP17 |
|  | Pr22 protein |
|  | p19 |
|  | stathmin 1 |
|  | Metablastin |
|  | LAP18 |
|  | Op18 |
|  | Leukemia-associated cytosolic phosphoprotein stathmin |
|  | pp19 |
|  | leukemia-associated gene |


---

|  |  |
| --- | --- |
| GO Component | cytosol |
|  | kinesin complex |
|  | microtubule |
|  | cytoplasm |


---

|  |  |
| --- | --- |
| GO ID | GO:0005874 |
|  | GO:0007019 |
|  | GO:0005737 |
|  | GO:0007409 |
|  | GO:0015631 |
|  | GO:0005515 |
|  | GO:0051272 |
|  | GO:0005829 |
|  | GO:0007052 |
|  | GO:0004871 |
|  | GO:0007017 |
|  | GO:0007242 |
|  | GO:0031115 |
|  | GO:0005871 |


---

|  |  |
| --- | --- |
| MIM | MIM:151442 |


---

|  |  |
| --- | --- |
| Connectivity | 302 |


---

|  |  |
| --- | --- |
| Entrez ID | 16765 |
|  | 3925 |
|  | 29332 |


---

|  |  |
| --- | --- |
| Agilent ID | A\_44\_P418920 |
|  | A\_14\_P123514 |
|  | A\_53\_P158929 |
|  | A\_43\_P11845 |
|  | A\_44\_P401165 |
|  | A\_14\_P129783 |
|  | A\_53\_P122894 |
|  | A\_14\_P133966 |
|  | A\_23\_P200866 |
|  | A\_52\_P319161 |
|  | A\_51\_P495641 |


---

|  |  |
| --- | --- |
| Cellular Localization | Microtubule |
|  | Cytoplasm |
|  | Cytosol |
|  | Cytoskeleton |
|  | Cell |
|  | Organelle |


---

|  |  |
| --- | --- |
| DbXref | KEGG pathway##04010##MAPK signaling pathway##http://www.genome.jp/dbget-bin/show\_pathway?rno04010+29332 |
|  | KEGG pathway##04010##MAPK signaling pathway##http://www.genome.jp/dbget-bin/show\_pathway?hsa04010+3925 |
|  | KEGG pathway##04010##MAPK signaling pathway##http://www.genome.jp/dbget-bin/show\_pathway?mmu04010+16765 |


---

|  |  |
| --- | --- |
| Pathway | Zn xs inventory |
|  | Zn xs DIN |


---

|  |  |
| --- | --- |
| GO Process | axonogenesis |
|  | microtubule-based process |
|  | negative regulation of microtubule polymerization |
|  | microtubule depolymerization |
|  | intracellular signaling cascade |
|  | mitotic spindle organization and biogenesis |
|  | positive regulation of cell motility |


---

|  |  |
| --- | --- |
| UniGene | Hs.209983 |
|  | Rn.555 |
|  | Mm.378957 |


---

|  |  |
| --- | --- |
| Affymetrix Probeset ID | 1386857\_at |
|  | 1415849\_s\_at |
|  | 1448113\_at |
|  | 1782\_s\_at |
|  | 200783\_s\_at |
|  | 217714\_3p\_x\_at |
|  | 217714\_x\_at |
|  | 70485\_at |
|  | 97909\_at |
|  | 97910\_at |
|  | g13518023\_3p\_a\_at |
|  | Hs.317661.0.S1\_3p\_x\_at |
|  | M31303\_rna1\_at |
|  | Msa.16998.0\_f\_at |
|  | Msa.17280.0\_f\_at |
|  | rc\_AI231821\_at |
|  | z31235\_f\_at |
|  | 63705\_f\_at |
|  | 63906\_f\_at |
|  | RC\_D51215\_f\_at |
|  | RC\_D51276\_f\_at |
|  | TC14392\_f\_at |
|  | TC14393\_f\_at |
|  | TC14393\_i\_at |
|  | TC35830\_at |
|  | TC35935\_at |


---

|  |  |
| --- | --- |
| GO Function | protein binding |
|  | tubulin binding |
|  | signal transducer activity |


---

|  |  |
| --- | --- |
| Nucleotide | NM\_203401 |
|  | AK014108 |
|  | BF686314 |
|  | X53305 |
|  | AK012347 |
|  | BC014353 |
|  | X94915 |
|  | NM\_017166 |
|  | BQ428400 |
|  | BC010581 |
|  | X94914 |
|  | AB064953 |
|  | L20255 |
|  | BQ772197 |
|  | NM\_203399 |
|  | L20257 |
|  | BC054396 |
|  | J04979 |
|  | M31303 |
|  | D28428 |
|  | X94912 |
|  | BC062234 |
|  | L20256 |
|  | M27876 |
|  | AL033528 |
|  | J04991 |
|  | AK013302 |
|  | BC082228 |
|  | AK002606 |
|  | BX647885 |
|  | L20258 |
|  | BM563894 |
|  | NM\_005563 |
|  | BC023313 |
|  | Z11566 |
|  | BC031831 |
|  | AK088628 |
|  | NM\_019641 |
|  | AK012231 |
|  | BG030679 |
|  | AK054594 |


---

|  |  |
| --- | --- |
| Protein | CAA64400 |
|  | BAB22225 |
|  | NP\_981944 |
|  | CAA77660 |
|  | P54227 |
|  | AAA42184 |
|  | BAC40463 |
|  | AAH82228 |
|  | BAB28778 |
|  | P13668 |
|  | AAH14353 |
|  | NP\_981946 |
|  | CAA37391 |
|  | BAB61894 |
|  | AAA81570 |
|  | BAB28109 |
|  | AAH54396 |
|  | AAH10581 |
|  | P16949 |
|  | BAB29161 |
|  | BAB28178 |
|  | CAA64401 |
|  | AAH62234 |
|  | BAA05794 |
|  | CAA64398 |
|  | AAH31831 |
|  | AAA59971 |
|  | NP\_058862 |
|  | AAA59980 |
|  | NP\_005554 |
|  | NP\_062615 |


---

|  |  |
| --- | --- |
| Organism | Mammal |


---

|  |  |
| --- | --- |
| Location | 4 65.7 cM (Mus musculus) |
|  | chromosome 4, 4 65.7 cM, 4 D3 (Mus musculus) |
|  | chromosome 5, 5q36 (Rattus norvegicus) |
|  | chromosome 1, 1p36.1-p35 (Homo sapiens) |


---

|  |  |
| --- | --- |
